# Meta-analysis of several gene lists for distinct types of cancer: A simple way to reveal common prognostic markers

**DOI:** 10.1186/1471-2105-8-118

**Published:** 2007-04-06

**Authors:** Xinan Yang, Xiao Sun

**Affiliations:** 1 State Key Laboratory of Bioelectronics, Southeast University, 210096 Nanjing, P.R.China

## Abstract

**Background:**

Although prognostic biomarkers specific for particular cancers have been discovered, microarray analysis of gene expression profiles, supported by integrative analysis algorithms, helps to identify common factors in molecular oncology. Similarities of Ordered Gene Lists (SOGL) is a recently proposed approach to meta-analysis suitable for identifying features shared by two data sets. Here we extend the idea of SOGL to the detection of significant prognostic marker genes from microarrays of multiple data sets. Three data sets for leukemia and the other six for different solid tumors are used to demonstrate our method, using established statistical techniques.

**Results:**

We describe a set of significantly similar ordered gene lists, representing outcome comparisons for distinct types of cancer. This kind of similarity could improve the diagnostic accuracies of individual studies when SOGL is incorporated into the support vector machine algorithm. In particular, we investigate the similarities among three ordered gene lists pertaining to mesothelioma survival, prostate recurrence and glioma survival. The similarity-driving genes are related to the outcomes of patients with lung cancer with a hazard ratio of 4.47 (p = 0.035). Many of these genes are involved in breakdown of EMC proteins regulating angiogenesis, and may be used for further research on prognostic markers and molecular targets of gene therapy for cancers.

**Conclusion:**

The proposed method and its application show the potential of such meta-analyses in clinical studies of gene expression profiles.

## Background

Changes in gene expression levels could reflect clinically distinct conditions. Genome-wide perspectives of gene expression can now be obtained, and these can be combined with other currently-used criteria to identify predictors of clinical outcome for specific cancers [1-7]. Also, distinct gene expression profiles can reportedly determine molecular treatment responses, e.g. in cancer [8]. Thus it is possible to discover biomarkers from gene expression profiles that help to predict outcomes, and this emphasizes the need in biomedical research to combine results from similar experiments in order to identify diagnostic or prognostic disease markers.

Much recent research has confirmed that microarray results are comparable among different laboratories, especially when a common platform and a set of procedures are used [9-13]. Integrative analysis that evaluates cancer transcriptome data in the context of data from other sources has received attention recently (reviewed by Rhodes and Chinnaiyan [14]). An important emerging argument concerns the uniformity of cancer metastases as well as the evolution of malignancy in primary tumors [15-17]. Grutzmann et al. ran meta-analysis on four studies for pancreatic cancer, and validated their identified signatures using RT-PCR and immunohistochemistry [13]. In particular, Glinsky and colleagues innovatively published a 11-gene signature that is displayed consistently in stem cells self-renewal pathways, and this is a powerful predictor for prognosis in 11 distinct types of cancer [17]. These results exemplify the clinical application of meta-analysis signatures detected in different cancer stages or types. Rhodes et al. [18] presented a comprehensive investigation of 40 data sets. They identified a robust signature of a set of differentially expressed genes when cancer and normal tissues were compared. A recent study [19] identified lists of differentially regulated genes that also significantly overlap with genes regulated by the tumor suppressors p16 and pRB. This work helps to translate genome-wide expression analyses into clinically useful cancer markers. Meta-analysis is a powerful tool for identification and validation marker genes in above studies [13,18]. However, in these studies, meta-signatures are identified on the basis of the individual genes used for analysis. Segal et al. [20] divided genes into sets and reported that certain sets show coherent behavior across a diverse group of clinical conditions. Another recent publication compared gene expression in two conditions to generate a gene list for each study, and then detected significant Similarities of Ordered Gene Lists (SOGL) [21] from different studies. The above two approaches extend the determination of significance from single study analysis to meta-analysis.

However, none of the above studies involving multiple cancers mentions independent prediction, which is a key bridge between molecular knowledge and clinical application. In particular, the SOGL approach can detect similarities between two gene lists, irrespective of significant differences between them, because it does not rely on differential gene expression in each single list having strong effects, but rather on consistent changes across multiple lists. SOGL is similar to other non-parameter statistical tests, except that it uses different weighting schemes for ranks. The ideal is to give higher weights to the genes which expressed more differentially, and to sum all the weighted orders to quantify the similarity. This approach allows the significance of similarity to be decided during meta-analysis and identifies the genes responsible for the similarity. In contrast to previous methods, SOGL does not depend on the definition of a particular "significance" threshold for a single study. Thus it is superior to other methods for detecting signatures in studies with weak effects or small sample sizes.

However, the similarities among gene lists are not guaranteed to be transferable [21]. With the discovery of common cancer signatures, there is a need to extend the method to several rather than two lists. Therefore, to meta-analyze many microarray profiles together, and to analyze the problem of outcome in highly noisy data, we have developed and implemented the SOGL method in this paper, extending it from the comparison of two gene lists to the comparison of multiple gene lists, which is useful for meta-analysis of microarray data. When the gene lists show similarity, we ask whether the similarity-driving genes improve the predictive power of a single study. To this end, we implement SOGL in two ways. One is to compare the accuracy of prediction by meta-analysis with that of individual analysis, which has already been successfully demonstrated for multiple cancer microarray data sets [11]. The other is to compare the traditional classical highest t-score with SOGL in selecting variables for classification, which has not been used in the context of cross-validation and class prediction. Finally, we discuss the predictive capacity of the similarity-driving genes detected in three solid tumors, and prove its success on another independent cancer data set.

## Results

Our major aim was to identify biological mechanisms, common to different kinds of cancer that involve genes and gene expression changes inducing poor outcomes, e.g. metastasis, recurrence and short-term survival. We assumed that such mechanisms may be revealed by gene expression profiles. We collected nine recently-published microarray data sets related to clinical outcomes (for details see Table 1). For meta-analysis, we developed SOGL from a test for two gene lists to a test for multiple gene lists, since the similarities among gene lists are not guaranteed to be transferable. In this section, we first performed a meta-analysis allowing common samples across data sets to generate artificial similarity and to identify it using SOGL. Then we turned to six data sets on solid tumor for discovery of similarity and its contributing genes. All the data sets were pre-processed independently for background correction, normalization, summarization and quality assessment using an Affymetrix platform pre-processing protocol. We adopted the methods for stabilizing variance to normalize these raw profiling files on an additive scale in the nine collected data sets, using the R package *compdiagTools*.

**Table 1:** Clinical information about the microarray studies we collected

Studies			samples with outcome notation			
study ID	cancer	#sample	N	#good	#poor	ratio

A [3]	breast	37	37	19	18	0.49
B [3]	breast	52	52	34	18	0.35
L [27]	lung	203	126	117	9	0.07
M [5]	mesothelioma	31	17	8	9	0.47
P [38]	prostate	102	21	13	8	0.38
G [65]	glioma	42	18	8	10	0.44
L1 [64]	T-cell leukemia	30	13	7	6	0.46
L1 [22]	pediatric leukemia	132	93	71	12	0.13
L2 [23]	pediatric leukemia	327	245	201	44	0.18

### Using SOGL on Leukemia studies

The data set described by Ross [22] used a relatively newly designed microarray platform with 132 representative cases from another data set with 327 cases [23]. Therefore a significant similarity between the gene lists generated from these two data sets were expected. Adding Another data set on leukemia outcome, we applied SOGL to comparison of more than two gene lists. Thus we performed the meta-analysis allowing partially common samples to generate an "artificial" similarity. However, finding a similarity in gene lists between samples run on different platforms is not our interest as many programs would find this. The question we addressed here is to evaluate whether our method improves the accuracy of prediction from individual studies when there is significant similarity.

**Figure 1:**
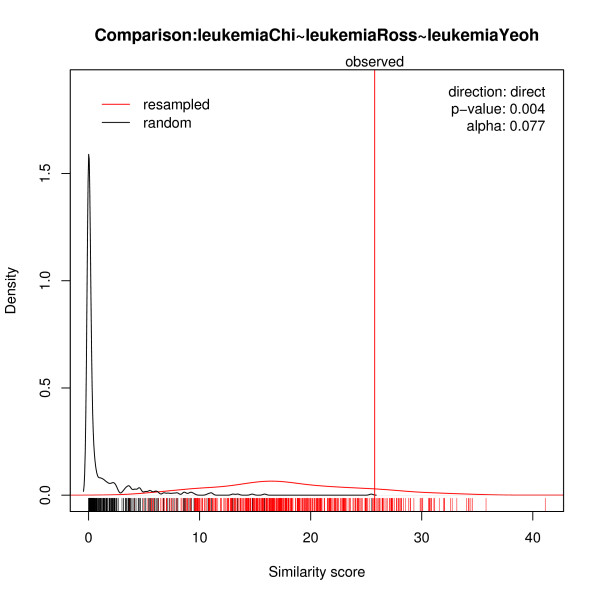
**Similarity scores for leukemia outcome**. The similarity of three gene orders for leukemia studies. In the plot, the red curve corresponds to estimated scores and the black curve to simulated random scores. These are kernel density estimates of the two-score distributions underlying the pAUC-score for optimal α*. The vertical red line denotes the observed similarity score. The bottom rugs mark the simulated values.

First, we separately analyzed all the data sets for differential gene expression between the good and the poor outcome groups. Differential expression was quantified using fold change and z-statistic respectively, and the result was used as the effect size for meta-analysis [24]. The later measurement is a moderated t-score with a fudge factor [25] and is expected to be more reliable. The significance of differential expression of a gene between outcome groups was estimated by comparison with the sizes of random effects in perturbed data. None of the three studies individually displayed strong evidence for differential expression; but while individual studies failed to identify signatures that might reliably distinguish between conditions, meta-analysis succeeded. All data sets ordered the genes, each beginning with the most markedly up-regulated genes in the poor outcome group and ending with the most markedly down-regulated ones. Matching of the probe sets between Affymetrix Hgu95av2 and Hgu133a, resulted in 10507 best-matched transcripts. These gene expression profiles revealed significant similarity in the outcome conditions of the three leukemia studies. Figure 1 shows the significance of this similarity. An empirical p-value = 0.004 (permutation times B = 1000, each based on permutation of gene ranks to estimate random similarity scores) was detected for an optimal α* which focused only on the first 150 genes in the orders. The significant similarity (p-value = 0.002) could also be observed when our method focused on the first 100 genes in the orders using z-statistic as effect size.

**Figure 2:**
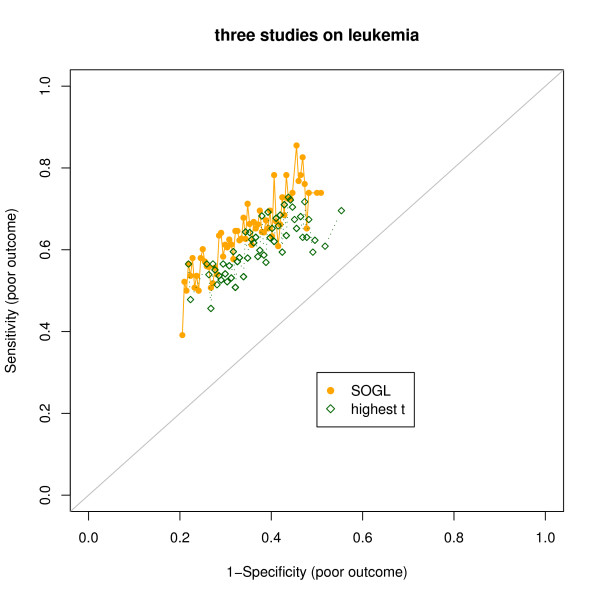
**Comparison of methods using leukemia data**. The ROC points for 500 prediction runs. Two points are generated for each time: the solid circle is the result from SOGL, and the diamond is the result from the same number of highest t-scores.

This led us to expect that variable selection by SOGL would improve the predictive capacity when the gene orders are significantly similar. For each subset of samples, we kept the number of transcripts selected by the highest t-scores exactly the same as the number of major intersection transcripts identified by SOGL method, while letting *α *= 0.015 to count the highest and lowest 750 items in the sets. The range of genes in common between the sets reflects the degree of similarity. For the comparison of gene orders in the three leukemia outcomes, we iterated 3-fold cross-validation together with support vector machine (SVM) algorithm D (= 500) times. The 75th and 25th percentiles of the numbers of selected genes are 75 and 48. The median is 61. Any increase in sensitivity will be accompanied by decrease in specificity, so to evaluate the predictive accuracy of the SOGL-selected genes and that of the highest traditional t-score, we drew ROC curves for both comparisons. Figure 2 shows the ROC curves generated from the leukemia studies. The SOGL curve follows the left-hand border and then the top border of the ROC space more closely, suggesting that the test is more accurate. In contrast, the highest t-score curve comes closer to the 45-degree diagonal of the ROC space, implying a less accurate test. The area under the ROC curve (AUC) is 0.73 (95% confidence interval (CI) 0.64–0.76) for SOGL using z-statistic as effect size, while 0.69 (95% CI 0.63–0.71) for the highest t-score, indicating SOGL tends toward more accurate than the highest t-score if gene lists are significant similar. In the same way, we observed no different AUC between the results of SOGL using fold changed effect size and highest t-score, that was 0.64 (95% CI 0.64–0.68) for SOGL, 0.63 (95% CI 0.57–0.69) for the highest t-score, suggesting that the improvement of prediction by SOGL is limited to highly significant similarity.

### Study on different solid tumors

**Figure 3:**
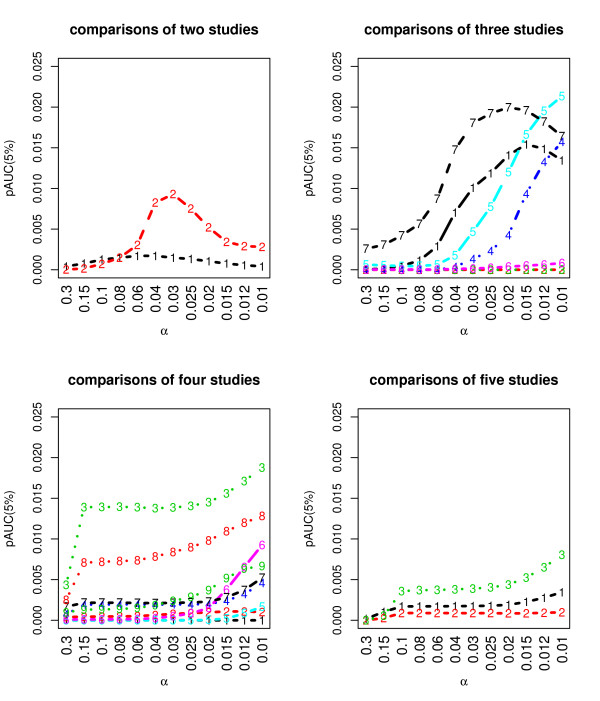
**Similar comparisons among 6 solid tumors**. 21 comparisons of gene lists show similar with separation between signal and noise. In the plot, *α *is given on the x axis and the pAUC-score for the randomized and alternative scores on the y axis. The pAUC test detects the difference between the distributions of alternative scores and random scores to select an optimized α*, which reaches a highest value for each comparison. We iterate a sub-sample strategy C (= 500) times to obtain an estimation of the variability of the similarity score and the random score. Each time, by bootstrapping 80% the labels of patients, we obtain the alternative effect size (signals). And by shuffling these labels of patients, we calculate the background noise of the same size. The details of the similarities are given in Table 4.

We then set out to determine the significant similarity among gene lists of different tumor outcomes. We needed confirmation first that the clinical diagnostic problem addressed here in regard to different kinds of cancer achieves similarity and improves the accuracy of prediction. To address this problem, we investigated six gene lists for comparing cancer outcomes, which are labeled A, B, L, M, P and G in Table 1. Figure 3 shows that 21 of the 57 possible comparisons from these gene lists show significant (*p *< 0.05) similarity for a pre-defined finite grid of parameter choices *α *∈ [0.3, 0.01] spanning the leading 400–1500 items in order. These comparisons include the gene lists referring to:

• Recurrence of breast cancer and lymph node status of breast cancer;

• The same two lists, and neuroendocrine of lung cancer;

• Survival of mesothelioma and glioma, and recurrence of prostate;

• The above set of lists and the lymph node status of breast cancer or neuroendocrine of lung cancer;

• and others.

All the above sets of gene lists achieved higher pAUC (partial area under curve) scores [26] than most other comparisons. A pAUC-score evaluates the degree of overlap between two distributions. Note that a higher pAUC-score shows a greater likelihood that the estimated SOGL scores exceed chance in our method, and a larger *α *indicates more similarities at the higher ends of the gene lists. This finding supports the emerging notion that when prognosis is poor, there are commonalities among distinct types of cancer in the dysregulation of gene expression, implying that poor prognosis is sometimes independent of the original cancer type. In contrast, this kind of similarity was not so significant when more than 4 of the studies we collected were compared, demonstrating that the similarities spanning tumor tissues are limited.

### The similarity among gene lists for glioma, prostate and mesothelioma outcomes

**Figure 4:**
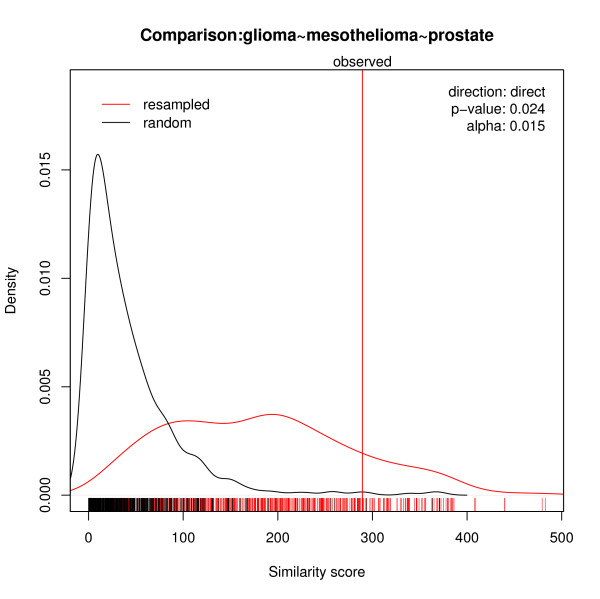
**Similarity score for solid tumors**. The similarities among three gene orders for different solid tumors. In the plot, the red curve corresponds to simulated observed scores and the black curve to simulated random scores. These are kernel density estimates of the two score distributions underlying the pAUC-score for optimal α*. The vertical red line denotes the observed similarity score. The bottom rugs mark the simulated values.

**Figure 5:**
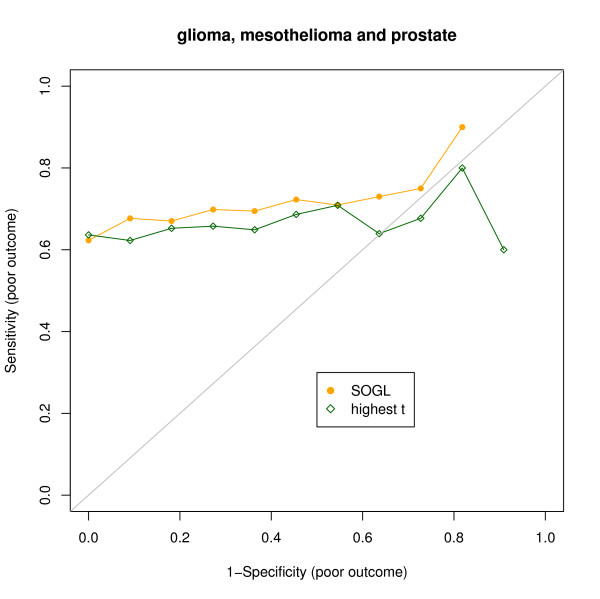
**Comparison of methods using solid tumor data**. The ROC points for 500 prediction runs. Each time, the solid circle is the result from SOGL, and the diamond is the result from the same number of highest t-scores.

Comparison of the ordered gene lists generated from the outcomes for glioma, prostate and mesothelioma typically shows significant similarity; and significance (B = 1000, *p *< 0.05) is found for all the pre-defined finite grids of observed orderings [100, 1500]. It means that even for the highest orderings (biggest *α *values), the numbers of genes common to these three orders are not due to chance. Figure 4 shows the significance of the similarity. An empirical p-value = 0.024 is obtained for an optimal α* focus on the highest 750 items in the order. To compare the accuracy of prediction by using SOGL as variable selection method to traditional highest-t-statistic, we iterated 3-fold cross validation D (= 500) times. The resulting 75th and 25th percentiles of the number of selected transcripts are 35 and 20. The median is 26. The superiority of SOGL is observed when the three solid tumor studies are integrated (for details see Figure 5). The area under the ROC curve is 0.747 (95% CI 0.709–0.774) for SOGL, 0.665 (95% CI 0.634–0.702) for the highest t-score. This proves that adopting SOGL for variable selection improves the predictive capacity when the gene lists involved are significantly similar. A similar improvement was observed when we examined a range of observed orderings [100, 1500].

We then turned to investigate the genes contributing to this similarity that were relevant to the survival of mesothelioma and glioma and the recurrence of prostate cancer. Table 2 shows the ranks and the symbols of these similarity-driving genes. The definition of "effect size" will affect the SOGL results and the identified genes. The genes identified by fold-change as effect size of SOGL yielded in 17 transcripts; 5 transcripts were reported if a moderated t-score with a fudge factor (also called as z-statistic) [25] was adopted as SOGL effect size. Four of these were identified by both approaches. Since our fold-change statistic is based on variance-stabilized data, it should generate a result similar to the t-statistic.

**Table 2:** The similarity-driving genes found in the G, M, and P studies

gene		rank(fold-change)			rank(z-statistic)		
Symbol	probeID	G	M	P	G	M	P

IGFBP3	37319_at	-11	-41	-11	-68	-53	-22
	1586_at	-35	-177	-52	-90	-128	-19
COL4A2	36659_at	-27	-4	-111			
COL4A1	39333_at	-17	-11	-58			
COL1A2	32306_g_at	-23	-86	-1			
PTGDS	38406_f_at	24	62	55			
	216_at	28	87	50	446	407	9
	38407_r_at	34	196	52			
ANXA2	769_s_at	-28	-16	-57			
ANXA2P3	31444_s_at	-31	-17	-72			
CPE	36606_at	23	147	137			
FN1	31719_at	-39	-61	-53			
BGN	38126_at	-34	-113	-41			
MDK	577_at	-85	-63	-37			
	38124_at	-96	-50	-78			
COL5A2	38420_at	-80	-51	-151	-84	-227	-30
POSTN	1451_s_at	-130	-52	-56			
PTTG1	40412_at				-69	-234	-86

A negative value indicates a relatively high expression level in the poor-outcome patients than in the good-outcome patients, and a positive value indicates a relatively low expression level in the poor-outcome patients.

Nevertheless, the z-statistic puts less weight on variances than a classical t-statistic. These genes contain a high proportion of known prognostic marker genes and represent biological processes involved in tumor progression and metastasis. To evaluate over-representation of GO annotations from gene lists that were calculated from specific microarray (Affymetrix Hgu95av2), we ran hypergeometric tests to compute p-values. It evaluates the likelihood that the corresponding number of annotations is occurring in a random list of genes of the same size. Interestingly, 4 of them are genes for the human extracellular matrix (ECM)-receptor interaction pathway (hypergeometric test p = 1e-6), namely COL4A1, COL1A2, COL5A2 and FN1. Moreover, 7 of our short-list of 13 genes encode ECM proteins and regulators of ECM assembly, namely FN1, BGN, POSTN, COL4A1, COL11A1, COL1A2 and COL5A2. The other 5 genes have roles in angiogenesis: ANXA2, CPE, MDK, IGFBP3, and 3 transcripts of PTGDS. Although ANXA2 (annexin A2) is a substrate for a variety of protein kinases, and plays an important role in plasmin regulation and in cancer cell invasiveness and metastasis, ANXA2P3 (annexin A2 pseudogene 3) is a novel marker not being previously reported. We discuss these genes in more detail in a later section.

### Validation of similarity-driving genes in the outcomes of three cancers on lung cancer data

**Figure 6:**
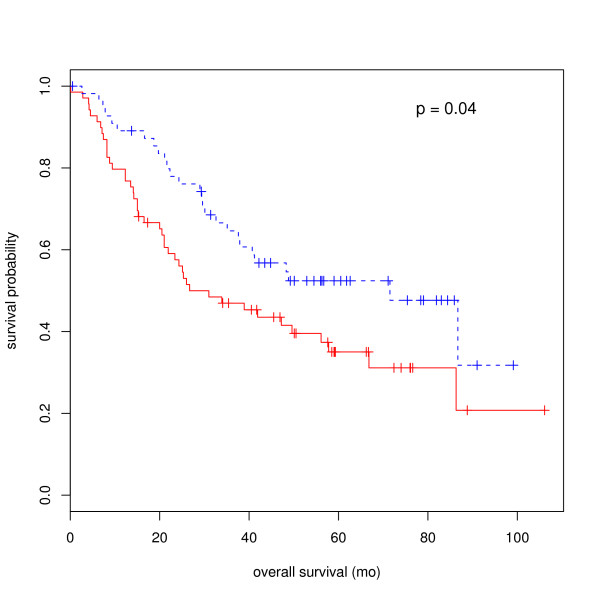
**Survival analysis of lung cancer patients**. Kaplan-Meier survival analysis of individual outcomes defined by 5 similarity-driving transcripts in the three solid tumors.

We have found that the neuroendocrine differentiation was significantly similar to the three gene lists M, P and G (comparison ID 4_9 in the Table 4 and Figure 3). And Bhattacharjee et al. reported that the C2 neuroendocrine differentiation was associated with good outcome [27]. We therefore tried to establish the utility of the 5-transcript signature for M-P-G similarity on the outcome of patients with lung cancer. We expected that the 5-transcript signature was related to the lung cancer interpreted as neuroendocrine if its change statistically relevants to cancer development. On the other hand, we did not expect a strong power to predict the outcomes, because many non-C2 adenocarcinoma patients have short survival times. To this end, we divided the 125 lung cancer patients into two outcome groups. We employed a robust K-means classification method, *Pam *[28] calling R package *cluster*, to partitions (clusters) the data into 2 clusters around medians using the 5-transcript signature. We then used a Cox proportional hazards regression model (calling the R package *survival*) to explore the relationship between the pam-predicted conditions and clinical survival. The estimated hazard ratio defined by our 5-transcript signature was 4.47 (p = 0.035). As Figure 6 shows, the median survival after therapy in the poor-prognosis subgroup was 26.7 months, compared to 71.5 months for patients in the good-prognosis subgroup. The estimated hazard ratio generated by the 17 transcripts was not significant (p = 0.25). It might due to the sub-optimal measurement of fold-change for gene expression studies. Here we relied on the remaining default settings of the R-package *cluster *and *survival*, though other classifier arguments may yield better results after sophisticated fine tuning. This result provides insights into the application of our microarray analysis in clinical settings and could help to identify novel targets for molecular pharmacodynamics.

**Table 4:** 21 Significantly similar comparisons of the ordered gene lists with the same labels used by the Figure 3

comparison ID	studies	*α*.opt	# up	#down	#genes (0.03)
2_1	A B	0.06	162	154	43
2_2	A P	0.03	166	164	46
3_1	A B L	0.015	267	176	16
3_2	A B M	0.3	142	271	8
3_3	A B P	0.012	135	252	4
3_4	A B G	0.01	140	255	7
3_5	A L P	0.01	198	213	12
3_6	B M G	0.01	0	169	5
3_7	M P G	0.02	206	187	17
4_1	A B L M	0.3	350	296	4
4_2	A B L P	0.012	0	340	0
4_3	A B L G	0.01	0	235	0
4_4	A B M G	0.01	314	395	7
4_5	A B P G	0.01	448	243	3
4_6	A L M P	0.01	483	354	6
4_7	A L M G	0.01	483	354	6
4_8	A M P G	0.01	463	251	7
4_9	L M P G	0.01	232	396	6
5_1	A B L M G	0.01	0	481	3
5_2	A B M P G	0.01	666	419	2
5_3	A L M P G	0.01	466	424	2

The column "# up" records how many orders to count for up-regulated genes [66], and "# down" records how many orders to count for down-regulated genes in poor outcomes.

We want to emphasize that we did not test the statistical significance of the identified genes with the survival outcomes by fitting the Cox proportional-hazards model to each gene [29]. We believe it contains information that the consensus change of these genes in a group, and this information is of critical importance in elucidating the complex genetic architecture of tumor progression, e.g. certain biochemical path. In fact, two of the small set of transcripts are insulin-like growth factor binding protein-3 (IGFBP3), over-expression of which has already annotated as apoptosis promoter of cancer cells, activated by p53 [30,31]. Moreover, it has recently been independently detected by other studies in vivo or in vitro that the increased expression of COL5A2 in colorectal cancer [32], the increased expression of PTTG1 with correlation to poor prognosis in glioma [33], and the down-regulation of the PTGDS as an important variable in liver and bladder cancer cell and in malignant progression forms of oral tissue [34-36].

## Discussion

Treatment of cancer patients is known to impact in several ways on prognosis. For an identical tumor, prognosis may be good if the condition has been diagnosed in good time but hopeless otherwise. Also, the set of genes that show significant changes of expression in one specific tumor includes genes that are significant for prognosis. Genes that are recognized statistically, especially in small data sets, might be of little value for new patients. In contrast, the genes that show consistent changes across all prognostic gene-lists have key roles in cancer development and progression. Therefore, to detect universal prognostic markers, integrated analysis based on large patient groups is required, and significance needs to be judged at the meta-analysis stage. SOGL quantifies and tests the similarities between two or more gene lists. The genes driving the similarity are those with prominent ranks in all the lists compared. Notwithstanding personal and other influences, these genes may genuinely indicate molecular alterations common among neoplasias. Another serious concern for bioinformatics researchers is the arbitrary or over-fitted choice of statistical approach that yields far-from-reliable gene sets. Information about clinical outcomes is unstable and weak because the differences among individuals might be large, and the challenge is to overcome this problem. Our results show that the SOGL method complements previous methods and is robust. The marker genes identified on the basis of one effect size concur with those based on another in our limited data. Though without strongly superiority, SOGL is tend to be more accurate than highest t-score for variable selection by meta-analysis. Studies that in isolation do not provide solid evidence for differential gene expression may present striking similarities in their gene lists. Thus SOGL can identify consensus signals from either strong or weak effects, independently of the arbitrary threshold. Moreover, it would be of greater interest to apply SOGL to the exploration of disease mechanisms based on these commonly changed genes in consensus. It is different from the approaches targeting only the "best" marker, result of SOGL might include genes that are so-called "redundant" by certain "threshold" of significance or correlation in individual study. Co-regulating genes in a biological path, genes in a parallel path, and genes having epistatic actions are in fact genes of critical importance in elucidating the complex genetic architecture of a complex disease [37]. Thus SOGL might be used to uncover the hidden pattern of genes on microarrays. Instead of distinctions of significance or correlation, it focuses on the genes relevant to the condition of interest that are consistently changed across multiple studies.

Biologists usually compare independent studies addressing the same research question to confirm findings. It is also possible to compare studies from slightly different but related contexts in order to discover common markers. This is an attempt to revolutionize cancer data sets to screen for common molecular features shared among phenotypically different types of cancer involving distinct biological underpinnings, disease progression, diagnosis and prognosis. We detected and confirmed that significant similarities span several kinds of cancer. This result supports the emerging notion that different types of tumors for which prognosis is poor share common disorders in the regulation of gene expression. This implies that poor prognosis sometimes develops independently of original cancer type.

A substantial literature suggests that the similarity-driving genes are promising as tumor markers and as targets for tumor therapy. The genes common to the top ends of the lists for the outcomes of the three cancers studied here include those originally used by Singh and Gordon [5,38] for outcome prediction, such as IGFBP3. FN1 has also been used in a real-time PCR-based multigene outcome predictive model for lymphoma [39] and prostate cancer [40]. Expression of POSTN is reportedly a bone metastasis from breast cancer [41] and is proposed as a prognostic marker in lung tumor invasion [42]. Dysregulation of ANXA2 has been reported in human bone cancer metastases [43] and is correlated with the clinical prognosis of prostate cancer [44]. Additional supportive evidence of the prognostic value of the genes in Table 2 from experiment in vitro and in vivo has been cited in the last section of result.

Our most striking finding, however, is the over-representation of genes detected from fold changes (MDK, CPE, POSTN, COL4A1, COL11A1, COL1A2, COL5A2, IGFBP3, FN1, ANXA2, BGN and PTGDS) and all 4 genes detected from the z-statistic as effect size (PTTG1, COL5A2, IGFBP3 and PTGDS) are associated with angiogenesis. Angiogenesis leads to the formation of a large anastomosing vascular network, allowing tumor growth, intravasation and the spread of metastases. MDK, which plays an important role in the intercellular interactions involved in angiogenesis, is reported to be strongly correlated with poor prognosis in a large number of cases irrespective of tissue type [45-50]. Another gene, CPE, is relatively down-regulated in the three poor-outcome samples of carcinoid tumors [51], and takes part in producing angiogenic factors upon the maturation of follicle stimulating hormone [52]. Generally, the breakdown of ECM proteins, which correlates with angiogenesis, is an essential step in cancer invasion and metastasis [53]. We found that up-regulation of 7 genes involved with the ECM is associated with poor cancer outcomes. ECM-related genes that promoted the strongest proliferation, including POSTN [54], BGN [55] type I collagen [56] and type IV collagen [56], have already been identified as cancer markers, and might be molecular targets for gene therapy. In addition, BGN and PTGDS have recently been reported in an in vitro angiogenesis system [57]. The oncogenic potential of PTTG1 has been well characterized in mouse fibroblast (NIH3T3) cells, in which it induces proliferation and promotes tumor formation and angiogenesis [58]. It has been reported as a prognostic marker for tumor invasiveness and metastasis [59] and is suggested to be a potent human oncogene [60]. These findings suggest that by inhibiting angiogenesis, it may be possible to restrict the blood supply to tumors and limit their ability to grow and metastasize. Our results support the anti-angiogenic hypothesis concerning polymeric FN1 [40] and ANXA2 [54] and suggest more candidate markers. Because the similarities among multiple tumor tissues can not be identified by speculation, we believe that further meta-analysis on more data will aid further research on prognostic markers of many cancers.

## Conclusion

For a small clinical trial, it is important to summarize all the evidence obtained and combine it with evidence from other trials or laboratory studies. Meta-analysis enables general conclusions to be drawn, develops support for hypotheses, and produces an estimate of the overall effects of a program, combining with the developed multiple statistical algorithm. This study suggests that our meta-analysis of gene lists for different clinical or physiological phenotypes provides a golden opportunity for detecting biologically relevant gene dysregulations between different phenotypes and possibly leading to improved diagnostic accuracy, or generating insightful molecular mechanisms to build the underlying bridges between different phenotypes. To this end, SOGL is superior to other measurements of gene selection for meta-analysis of clinical microarrays for handling study-to-study differences. It focuses on the genes relevant to the condition of interest that are consistently changed across multiple studies, rather than on distinctions of significance or correlation. Our study has assessed its potential for identifying prognostic markers of multiple cancer types from studies of different laboratories, especially for studies with large inter-individual variations or small sample size. The proposed method is a complementarity and enlargement algorithm for research on gene expression.

In addition, our results suggest and confirm that a common molecular mechanism underlies the poor outcomes of several kinds of cancer. The genes we detected have important implications for our understanding of the potential involvement of angiogenesis in the malignant progression of primary tumors. It suggests that meta-analysis has considerable potential in clinical studies of gene expression profiles, which is a focus of active research for computer-assisted diagnosis. To ensure reproducibility of our biological findings, larger numbers representing a greater percentage of disease is required. It is expected that further studies incorporating more data sets with larger number of samples will identify universal prognostic markers in cancer.

## Methods

### Transcript expression data and outcome

In transcriptional research, the raw data have to be corrected for different conditions by normalization. We normalized all raw profiling files on an additive scale by pre-processing methods for stabilizing variance [61]. "An additive scale" means transforming the intensities to a scale where the variance is approximately independent of the mean intensity. This can be achieved by calibrating for sample-to-sample variations through shifting and scaling, or by log-transforming the data. For simplicity, we focused on the published microarray studies of cancer outcomes based on Affymetrix chips, which have sufficient data and have gained acceptance in recent years because of the reliable annotation and identification and the good hybridization characteristics of oligonucleotides with wide-ranging expression levels [62]. Only the best-matched transcripts [63] were used to compare studies based on different chips.

The definitions of outcomes for all the studies we collected strictly followed those of the original papers. To evaluate the power of signature detection in transcript expression and the accuracy of prediction by our adopted method, we integrated all the relatively non-malignant outcomes as "good". In contrast, the patients were grouped as "poor" if they suffered shorter survival or if there was recurrence within the observed time. The data sets were:

• **Leukemia C: **The data came from research on adult T-cell acute lymphoblastic leukemia (ALL) [64]. The good prognosis group consisted of 7 patients in complete clinical remission (CCR) and 2 patients who had not relapsed within two years. The poor prognosis group consisted of 6 refractory patients and 12 who had relapsed within two years.

• **Leukemia Y: **The data included 327 children suffering leukemia [23]. Excluding the patients without outcome information, The good group consisted of 201 CCR patients, while the poor responder group consisted of 44 patients with different types of relapse.

• **Leukemia R: **93 patients with prognostic information from above study were examined the gene expression profiling by Ross et al. using another microarray chip [22]. The good prognosis group consisted of 71 CCR patients. The poor prognosis group consisted of 16 relapsed patients and six 2nd AML patients.

• **Mesothelioma: **A prognostic study on mesothelioma, a lethal neoplasia of the pleura [5]. The good responder group consisted of 8 patients who survived more than seventeen months, while the 10 patients in the poor responder group survived less than six months.

• **Prostate: **This comparison was constructed from 21 prostate tumor samples with respect to recurrence following surgery [38]. The good prognosis group consisted of 13 patients who had shown no relapse for at least four years, and the poor outcome groups consisted of 8 relapse patients.

• **Glioma: **This comparison was based on the data of Shai et al. [65]. The good prognosis group consisted of 8 primary (not secondary) glioblastoma multiforme (GBM) patients of various pathological types and grades with a survival time of more than three years, while the poor responder group consisted of 10 malignant glioma patients who survived less than one year.

• **Breast 1: **The data were taken from a prognostic study of primary breast tumors by Huang et al. [3]. In total, 37 patients were included. The good prognosis group consisted of 19 "low-risk" patients, and the poor responder group consisted of 18 patients identified as "high-risk" by their lymph-node status.

• **Breast 2: **These data were also described by Huang et al. [3]. Here, however, the prognostic groups were defined directly by clinical outcome. The good responder group consisted of 34 patients who were recurrence-free over three years, while the poor responder group consisted of 18 patients who suffered recurrent disease within the first three years after surgery.

• **Lung: **The data included 126 adenocarcinoma (one subtype of lung cancer) cases without metastases reported by Bhattacharjee et al. [27]. The lung cancer data set did not define the outcome classification for each case. However, the author reported that the neuroendocrine C2 adenocarcinoma were associated with a less favorable survival outcome. Therefore the poor responder group consisted of 9 neuroendocrine C2 adenocarcinoma patients, while the good responder group consisted of all the other 117 adenocarcinoma patients.

### Detecting similarities amongst ordered gene lists and their contributing genes

SOGL introduces a comparison between two states [21,66]. Preferably, one state relates to a good outcome or prognosis and the other to a bad outcome. Let D* be the collection of studies. Applying a standard statistic to each study, *d *∈ D*, we can obtain a gene list gid
 MathType@MTEF@5@5@+=feaafiart1ev1aaatCvAUfKttLearuWrP9MDH5MBPbIqV92AaeXatLxBI9gBaebbnrfifHhDYfgasaacH8akY=wiFfYdH8Gipec8Eeeu0xXdbba9frFj0=OqFfea0dXdd9vqai=hGuQ8kuc9pgc9s8qqaq=dirpe0xb9q8qiLsFr0=vr0=vr0dc8meaabaqaciaacaGaaeqabaqabeGadaaakeaacqWGNbWzdaqhaaWcbaGaemyAaKgabaGaemizaqgaaaaa@30DC@ representing the differences in expression between samples in the poor- and good-outcome classes. The original similarity score, *S*_*n*_, is based on the number of overlapping genes in the top n ranks deriving from *k *= 2 gene lists [21,66]. We can assign a more general similarity score to a comparison of several gene lists as Table 3 shows. Thus the extended SOGL score is here defined as a summation of weighted partial intersection sizes on *k *ends of ordered gene lists:

**Table 3:** Illustration of the cardinality of the intersection *O*_*n*_(*G*_*D*_)

n	*G*_1_	*G*_2_	*G*_3_	*O*_*n*_(*G*_1,2,3_)
1	a	**h**	**k**	0
2	**k**	w	z	0
3	**h**	b	**h**	1
4	m	**K**	b	2
5	**t**	a	**t**	2
6	w	**t**	i	3
...	...	...	...	...

Rows correspond to the orderings (n), columns to the different studies. *G*_*d*_(*n*) denotes the n'th most strongly up-regulated transcript in study *d *that changes when poor-outcome is compared with good-outcome.

Sα(G1,G2,...,Gk)=∑nwnαOn
 MathType@MTEF@5@5@+=feaafiart1ev1aaatCvAUfKttLearuWrP9MDH5MBPbIqV92AaeXatLxBI9gBaebbnrfifHhDYfgasaacH8akY=wiFfYdH8Gipec8Eeeu0xXdbba9frFj0=OqFfea0dXdd9vqai=hGuQ8kuc9pgc9s8qqaq=dirpe0xb9q8qiLsFr0=vr0=vr0dc8meaabaqaciaacaGaaeqabaqabeGadaaakeaacqWGtbWudaWgaaWcbaacciGae8xSdegabeaakiabcIcaOiabdEeahnaaBaaaleaacqaIXaqmaeqaaOGaeiilaWIaem4raC0aaSbaaSqaaiabikdaYaqabaGccqGGSaalcqGGUaGlcqGGUaGlcqGGUaGlcqGGSaalcqWGhbWrdaWgaaWcbaGaem4AaSgabeaakiabcMcaPiabg2da9maaqafabaGaem4DaC3aa0baaSqaaiabd6gaUbqaaiab=f7aHbaaaeaacqWGUbGBaeqaniabggHiLdGccqWGpbWtdaWgaaWcbaGaemOBa4gabeaakiabcYcaSaaa@4AAF@

where decreasing weights (w) are used as: wnα=e−αn
 MathType@MTEF@5@5@+=feaafiart1ev1aaatCvAUfKttLearuWrP9MDH5MBPbIqV92AaeXatLxBI9gBaebbnrfifHhDYfgasaacH8akY=wiFfYdH8Gipec8Eeeu0xXdbba9frFj0=OqFfea0dXdd9vqai=hGuQ8kuc9pgc9s8qqaq=dirpe0xb9q8qiLsFr0=vr0=vr0dc8meaabaqaciaacaGaaeqabaqabeGadaaakeaacqWG3bWDdaqhaaWcbaGaemOBa4gabaacciGae8xSdegaaOGaeyypa0Jaemyzau2aaWbaaSqabeaacqGHsislcqWFXoqycqWGUbGBaaaaaa@37D7@. In this way, we strengthen the two ends of the integrated transcript orders. By setting the parameter *α*, one can calibrate the weight to decide that how deeply these gene orders are to be investigated.

To calibrate an adaptive α* to the gene lists of interest, we partially (80%) resampled the class labels of patients in the original raw data [21,66]. Class-balanced resampling from the good- and poor-outcome groups estimates the signal (alternative score), and class-shuffled resampling in each study simulates background noise of the same size (noise score). This resampling for estimation step was iterated C (= 500) times. To evaluate the separation of these two score distributions, we applied the pAUC-score [26] resulting from a comparison of signal and noise. Fixing a maximally acceptable false positive rate *w*_0_, we measured separability as the area under ROC(*w*) with *w *<*w*_0 _as

paucαi(w0)=∫0w0ROC(w)dw,
 MathType@MTEF@5@5@+=feaafiart1ev1aaatCvAUfKttLearuWrP9MDH5MBPbIqV92AaeXatLxBI9gBaebbnrfifHhDYfgasaacH8akY=wiFfYdH8Gipec8Eeeu0xXdbba9frFj0=OqFfea0dXdd9vqai=hGuQ8kuc9pgc9s8qqaq=dirpe0xb9q8qiLsFr0=vr0=vr0dc8meaabaqaciaacaGaaeqabaqabeGadaaakeaacqWGWbaCcqWGHbqycqWG1bqDcqWGJbWydaWgaaWcbaacciGae8xSde2aaSbaaWqaaiabdMgaPbqabaaaleqaaOGaeiikaGIaem4DaC3aaSbaaSqaaiabicdaWaqabaGccqGGPaqkcqGH9aqpdaWdXaqaaiabbkfasjabb+eapjabboeadjabcIcaOiabdEha3jabcMcaPiabbsgaKjabdEha3bWcbaGaeGimaadabaGaem4DaC3aaSbaaWqaaiabicdaWaqabaaaniabgUIiYdGccqGGSaalaaa@4AC3@

where *w *was the false positive rate, and ROC(*w*) was the true positive rate. A high pAUC-score indicates good separation. Given a parameter *α*_*i*_, the separation of alternative scores and noise scores indicates the similarity between the leading genes in these gene orders. For a predefined finite grid of parameters, then we can pick the value providing the best discrimination between signal and noise. The significance was then evaluated for a given *α*. To this end, we simulated the distribution of similarity score under assumption of unrelated lists and generated B (= 1000) set of ternately random ranks to calculate the random scores. Significance was evaluated by computing an empirical p-value for the observed scores from the B random scores.

The similarity-driving genes should be consistently represented among the leading items in the gene orders. One can count a cutoff value n* such as ∑n=1n∗e−α∗nOn
 MathType@MTEF@5@5@+=feaafiart1ev1aaatCvAUfKttLearuWrP9MDH5MBPbIqV92AaeXatLxBI9gBaebbnrfifHhDYfgasaacH8akY=wiFfYdH8Gipec8Eeeu0xXdbba9frFj0=OqFfea0dXdd9vqai=hGuQ8kuc9pgc9s8qqaq=dirpe0xb9q8qiLsFr0=vr0=vr0dc8meaabaqaciaacaGaaeqabaqabeGadaaakeaadaaeWaqaaiabdwgaLnaaCaaaleqabaGaeyOeI0ccciGae8xSde2aaWbaaWqabeaacqGHxiIkaaWccqWGUbGBaaGccqWGpbWtdaWgaaWcbaGaemOBa4gabeaaaeaacqWGUbGBcqGH9aqpcqaIXaqmaeaacqWGUbGBdaahaaadbeqaaiabgEHiQaaaa0GaeyyeIuoaaaa@3DE2@ to accounts for 95% of the score *S*_α*_, given an identical α*. Note that SOGL is the sum of the scores for the two ends. Thus we identify the similarity scores for up- and down-regulation, ignoring genes for which the isolated up- or down-regulation yields scores no higher than the 99th percentile of the random scores. The expected random scores are given by B (= 1000) shuffled orderings. For example, if a certain significance is due to the most strongly down-regulated genes but not to the most strongly up-regulated genes, we ignore the intersection of up-regulated genes.

### Estimating the accuracy of prediction

We expected that combined studies will predict the outcome for single patients better than a single study can, assuming that there is commonality in the dysregulation of gene expression for certain malignant processes. To validate this assumption, we calculated the number of correct predictions for each study via two steps. (1) All patients from three similar studies were mixed into one integrated data set to cross-validate the outer and inner loops. This resulted in a vote matrix containing the number of times each sample was assigned to each class in the outer cross-validation loop. We counted the coincidences between true class and consensus class for samples study by study to obtain three tables. (2) The same cross-validation was run on single data to obtain independent tables for each study. For both steps, we repeated the cross-validation with the same stratified strategy (class-balanced folds [67]) and adopted the identified variable selection method and the classification method. We assume that we can combine data from different studies into one replicated data set, if the gene lists are significantly similar for a certain two-condition test.

We next compared SOGL with the traditional highest t-score to select variables for prediction, carrying out the same classification and patient clustering strategy before meta-analysis. To avoid study-to-study bias or prevalence of smaller sample sizes, we randomly employed class-balanced [67] and study-balanced training sets. "Class-balanced" means that we guarantee the combined training set comprises approximately half poor-outcome and half good-outcome patients. "Study-balance" means that we guarantee the training set contains all the different tumors, and keeps more or less the same proportion of each. Patients not used in the combined training set were used for validation. For SOGL variable selection, we focused on a fixed number of orderings to calculate the similarity score, and selected the intersection to account for 95% of the score. The resulting variables were used to predict the outcomes for patients in the associated validation set after tuning hyperparameters of SVM. After this step, we recorded the number of selected genes, then picked the same number of genes with the highest t-statistic to estimate the accuracy of prediction in the validation set. The above training/validation step was iterated D (= 500) times carrying SVM algorithm performing linear kernel by R package *e1071*. To compare the two variable-selection methods, we drew a Receiver Operating Characteristic (ROC) curve from the correct error metrics generated from the D repeats of training/validation step for each test. ROC is a plot of the true positive rate (TPR) on the y axis against the false positive rate (FPR) on the x axis for the different possible cut-off points of a diagnostic test. Thus, for every observed FPR, we calculated the mean value of the corresponding TPR to plot the point on the ROC curve. Let *u *be the good prognostic for the true good-outcome patients; *v*e the bad prognostic for the true good-outcome patients; *t *be the good prognostic for the true bad-outcome patients; and *s *be the bad prognostic for the true bad-outcome patients. For the null hypothesis that all patients are poor outcome, the sensitivity and specificity are:

*TPR*(*poor *- *outcome*) = *s/*(*s *+ *t*); *FPR*(*poor *- *outcome*) = *v*/(*u *+ *v*).

We measured the area under the ROC curves to evaluate the difference between SOGL and t-scores.

## Authors' contributions

XY carried out the data collection, performed the statistical analysis, and drafted the manuscript. XS participated in the design of the study. All authors read and approved the final manuscript.
